# Antihypertensive medication is not associated with increased disease severity in atopic dermatitis in the TREATgermany registry

**DOI:** 10.1111/ddg.15927

**Published:** 2025-10-23

**Authors:** Moritz M. Hollstein, Stephan Traidl, Thomas Werfel, Jochen Schmitt, the TREATgermany Study Group

**Affiliations:** ^1^ Department of Dermatology Venereology and Allergology University Medical Center Göttingen Göttingen Germany; ^2^ Department of Dermatology and Allergy Hannover Medical School Hannover Germany; ^3^ Center for Evidence‐Based Healthcare University Hospital Carl Gustav Carus and Carl Gustav Carus Faculty of Medicine Technische Universitaet Dresden Dresden Germany

**Keywords:** Antihypertensive medication, atopic dermatitis, disease severity, eczema, registry

Dear Editors,

Eczema may be of multifactorial origin. It is characterized by skin thickening, redness, scaling, and pruritus. Atopic dermatitis (AD) may be seen as the major cause,^1^ even though some cases are difficult to diagnose: Patients with intrinsic AD may have late disease onset, preserved skin barrier function, and have metal contact hypersensitivity.^2^ Drug intake may also cause eczema.^3^ Recently, Ye and colleagues found prescription of antihypertensive medication increased eczematous dermatitis diagnoses in patients of 60 years and above from 9 per 1,000 patient‐years at baseline to 11 to 12 per 1,000 patient‐years.^4^ In particular, diuretics (HR: 1.21, 95% CI: 1.19–1.24) and calcium channel blockers (CCBs) (HR: 1.16, 95% CI: 1.14–1.18) showed an association with eczematous lesions. To investigate the association of antihypertensive drugs and AD, we analyzed real‐life data from the TREATgermany registry, one of the largest AD registries worldwide.^5^, ^6^

## METHODS

TREATgermany, initiated in 2016, is a registry based on a prospective observational cohort study of patients with moderate to severe AD (ethics approval *EK TUD 118032016*, clinicaltrails.gov listing *NCT03057860*). Inclusion criteria, methods, and objectives have been published elsewhere.^6^ Data was extracted for adults (≥ 18 years) in March 2022. For the current analysis, the baseline visit data was used. Data including demographic information (e.g., age, gender), medication, and disease severity was used. Disease severity was assessed by *objective SCORing Atopic Dermatitis* (oSCORAD), *Eczema Area and Severity Index* (EASI), *patient's global assessment* (PGA), and *patient‐oriented eczema measure* (POEM). The *Dermatology Life Quality Index* (DLQI) was also assessed. Statistical analyses were conducted using the gtsummary (version 2.0.0) and ggplot2 (3.5.1) packages in R (4.2.1). Quantitative variables are presented as mean and standard deviation (SD).

## RESULTS

Of the registry patients, 618 were female (43.6%) and 799 (56.4%) were male (data available on request). Age ranged between 18 and 88 years and averaged 40.4 years (median: 28 years, Q1: 28 years, Q3: 52 years; Table 1). Average disease onset had been at 9.3 years of age. 210/1,417 (14.8%) patients were prescribed antihypertensive medication (Table 1). 150/1,417 (10.6%) were ≥ 60 years. In this subgroup, mean age was 66.3 and ranged between 60 and 88 years (median: 64 years, Q1: 62 years, Q3: 69 years; Table 1), and 71/150 (47.3%) received antihypertensive medication. In the adult cohort and the subgroup ≥ 60 years, hypertensive medication was not associated with increased physician‐reported disease severity (oSCORAD, EASI) or the patient‐reported outcome PGA (Table 2). In the overall cohort, but not in the subgroup, antihypertensive medication was associated with a significantly lower (i.e. better) DLQI and POEM (Table 2).

**TABLE 1:** Patient characteristics.

Characteristics		Antihypertensive Medication	
	Overall	No	Yes
	n = 1,417^1^	n = 1,207^1^	n = 210^1^
**BMI [kg/m^2^]**			
Mean (SD)	25.80 (5.13)	25.24 (4.71)	29.05 (6.21)
n non‐missing	1,399	1,193	206
Median, Q1, Q3	25, 22, 28	24, 22, 27	28, 25, 32
**Sex**			
Female	613 (43.5%)	551 (45.8%)	62 (30.1%)
Male	795 (56.5%)	651 (54.2%)	144 (69.9%)
Non‐missing	1,408	1,202	206
**Age [years]**			
Mean (SD)	40.32 (14.58)	37.72 (13.42)	55.27 (11.71)
n non‐missing	1,416	1,206	210
Median, Q1, Q3	38, 28, 52	35, 26, 48	56, 48, 62
Range	18 ‐ 88	18 ‐ 82	25 ‐ 88
**Age at AD onset [years]**			
Mean (SD)	9.31 (16.18)	7.23 (13.08)	21.20 (24.86)
n non‐missing	1,392	1,185	207
Median, Q1, Q3	2, 0,11	1, 0, 6	9, 0, 40

*Subgroup ≥ 60 years*			
	Overall n = 150^1^	No n = 79^1^	Yes n = 71^1^
**BMI [kg/m^2^]**			
Mean (SD)	26.28 (4.84)	25.31 (4.30)	27.37 (5.20)
n non‐missing	149	79	70
Median, Q1, Q3	25, 23, 29	24, 23, 27	27, 24, 30
**Sex**			
Female	55 (36.9%) 149 (99)	33 (41.8%) 79 (100)	22 (31.4%) 70 (99)
Male	94 (63.1%) 149 (99)	46 (58.2%) 79 (100)	48 (68.6%) 70 (99)
**Age [years]**			
Mean (SD)	66.31 (6.13)	65.37 (5.06)	67.35 (7.03)
n non‐missing	150	79	71
Median, Q1, Q3	64, 62, 69	63, 62, 69	65, 62, 72
Range	60 ‐ 88	60 ‐ 82	60 ‐ 88
**Age at AD onset [years]**			
Mean (SD)	30.59 (28.14)	24.81 (25.59)	36.94 (29.59)
n non‐missing	149	78	71
Median, Q1, Q3	30, 1, 58	14, 1, 53	40, 2, 65

*Abbr*.: AD, atopic dermatitis; BMI, body mass index; Q1, first quartile; Q3, third quartile; SD, standard deviation

^1^ n (%), n non‐missing (% non‐missing). Data entries with missing data on antihypertensive medication were excluded.

**TABLE 2:** Disease severity assessed using patient‐ and physician‐rated measurements and by antihypertensive medication intake.

Characteristics		Antihypertensive Medication		
	Overall	No	Yes	p‐value^2^
	n = 1,417^1^	n = 1,207^1^	n = 210^1^	
**Patient global assessment (PGA)**				0.087
Clear	33 (2.36%)	27 (2.26%)	6 (2.94%)	
Almost clear	123 (8.79%)	99 (8.28%)	24 (11.8%)	
Mild	279 (19.9%)	229 (19.2%)	50 (24.5%)	
Moderate	400 (28.6%)	349 (29.2%)	51 (25.0%)	
Severe	409 (29.2%)	361 (30.2%)	48 (23.5%)	
Very severe	155 (11.1%)	130 (10.9%)	25 (12.3%)	
n non‐missing	1,399	1,195	204	
**DLQI**				0.006
Mean (SD)	11.73 (7.83)	11.95 (7.77)	10.47 (8.07)	
n non‐missing	1,401	1,197	204	
**POEM**				0.001
Mean (SD)	16.63 (7.68)	16.91 (7.61)	14.96 (7.93)	
n non‐missing	1,399	1,195	204	
**EASI**				0.2
Mean (SD)	15.68 (12.78)	15.79 (12.74)	15.02 (13.04)	
n non‐missing	1,409	1,201	208	
**oSCORAD**				0.3
Mean (SD)	39.83 (16.55)	40.00 (16.26)	38.90 (18.16)	
n non‐missing	1,411	1,204	207	

*Subgroup ≥ 60 years*				
	Overall n = 150^1^	No n = 79^1^	Yes n = 71^1^	
**Patient global assessment (PGA)**				0.5
Clear	5 (3.38%)	2 (2.53%)	3 (4.35%)	
Almost clear	16 (10.8%)	5 (6.33%)	11 (15.9%)	
Mild	28 (18.9%)	17 (21.5%)	11 (15.9%)	
Moderate	38 (25.7%)	20 (25.3%)	18 (26.1%)	
Severe	40 (27.0%)	23 (29.1%)	17 (24.6%)	
Very severe	21 (14.2%)	12 (15.2%)	9 (13.0%)	
n non‐missing	148	79	69	
**DLQI**				0.9
Mean (SD)	10.18 (7.56)	10.13 (7.32)	10.23 (7.89)	
n non‐missing	148	79	69	
**POEM**				0.15
Mean (SD)	14.84 (8.37)	15.80 (8.46)	13.75 (8.20)	
n non‐missing	148	79	69	
**EASI**				0.7
Mean (SD)	16.56 (14.61)	17.36 (15.76)	15.64 (13.22)	
n non‐missing	148	79	69	
**oSCORAD**				0.9
Mean (SD)	40.50 (17.53)	41.15 (16.65)	39.75 (18.60)	
n non‐missing	147	79	68	

*Abbr*.: DLQI, dermatology life quality index; EASI, eczema area severity index; oSCORAD, objective scoring atopic dermatitis; PGA, patient global assessment; POEM, patient‐oriented eczema measure; SD, standard deviation

^1^ n (%), n non‐missing (% non‐missing). Data entries with missing data on antihypertensive medication were excluded.

^2^ p‐values were calculated using Wilcoxon rank‐sum tests (DLQI, POEM, EASI, oSCORAD) and Fisher's exact test (PGA).

## DISCUSSION

Prior to 2024, research suggested a possible association between calcium channel blocker (CCB) intake and eczematous dermatitis (supplementary Table S1). Recently, Ye and colleagues reported multiple antihypertensive drugs are associated with increased eczematous dermatitis incidence in patients ≥ 60 years: diuretic drugs, CCBs, ACE inhibitors, and β‐blockers.^4^ The effect size was the smallest for ACE inhibitors (HR, 1.02; 95% CI, 1.00–1.04). It remained unclear whether the associations were causal and clinically meaningful.

From the five Read codes (clinical codes formerly used in the UK National Health Service to record patient data in both primary and secondary care) analyzed by Ye and colleagues, several may be directly attributed to AD in patients ≥ 60 years: “Atopic dermatitis/eczema,” “Infantile eczema,” “Flexural eczema,” and “Allergic (intrinsic) eczema.” Statistically, many other “Eczema NOS” cases may likewise be attributed to AD. Based on a cross‐sectional analysis of 203,813 participants in the All of Us research program, 51% of eczema cases in adolescents were classified as AD.^1^

Our analyses indicate that antihypertensive medication was not associated with increased disease severity in the full (adult) AD cohort or in the subgroup ≥ 60 years. In a different cohort of heart failure patients, our group was also able to show that pruritus, a major symptom of eczema, was not associated with antihypertensive medication.^7^ Despite a much smaller sample size in both analyses, these data point towards a negligible clinical relevance of antihypertensive medication in disease severity. From a theoretical standpoint, a causal relationship between eczema and antihypertensive medication would be surprising considering the different pharmacological activities of antihypertensive drugs. Specifically in the case of CCBs, a potential pathomechanism has been proposed.^4^ CCB‐induced iron uptake and retention in human keratinocytes may cause apoptosis and spongiosis.

These changes may clinically and histomorphologically resemble eczema. When comparing the presented results extracted from the TREATgermany cohort with the findings from Ye et al., the following should be acknowledged: First, TREATgermany does not register mild AD cases. Second, in TREATgermany, AD was diagnosed by dermatologists, while Ye et al. analyzed eczema read codes from primary care. Limitations of the present analyses can be seen in the exclusive use of baseline data, potentially overlooking changes in medication and disease status over time. In addition, relatively small number of patients in the subgroups and the study design restrict causal inferences and generalizability.

In conclusion, in the TREATgermany cohort antihypertensive drugs were not associated with increased disease severity.

## FUNDING

TREATgermany is an academic, investigator‐initiated clinical disease registry that is financially supported by AbbVie Deutschland GmbH & Co. KG, Almirall Hermal GmbH, Galderma S.A., LEO Pharma GmbH, Lilly Deutschland GmbH, Pfizer Inc. and Sanofi.

## CONFLICT OF INTEREST STATEMENT

J.S. reports institutional grants for investigator‐initiated research from the German Federal Joint Committee, German Ministry of Health, German Ministry of Research, European Union, German Federal State of Saxony, Novartis, Sanofi, ALK, and Pfizer. He participated in advisory board meetings as a paid consultant for Sanofi, Lilly, and ALK. J.S. serves the German Ministry of Health as a member of the German National Council for Health and Care. S.T. and M.H. declare no conflict of interest. T.W. has received honoraria for lectures or scientific advice on atopic dermatitis from AbbVie, Almirall, Galderma, Janssen/JNJ, LEO Pharma, Leti, Lilly, Novartis, Pfizer, and Regeneron/Sanofi.

## Supporting information



Supplementary information
