# Supplementary material for: Antihypertensive medication is not associated with increased disease severity in atopic dermatitis in the TREATgermany registry
Source: J Dtsch Dermatol Ges. 2025 Oct 23;24(2):252–5. doi: 10.1111/ddg.15927 (PMC12875170; doi:10.1111/ddg.15927)
Supplement: Supplementary file 1 — Supplementary information [file DDG-24-252-s001.docx]

**Supplements**

The TREATgermany Study Group is led by the PSs J Schmitt / Dresden, S Weidinger / Kiel and T Werfel / Hannover and consists of the recruiting centres named in the authors list and the following recruiting centres: M Hilgers, Clinics for Dermatology and Allergy, University Hospital Aachen, Aachen, Germany/ M Bell, Practice Dr. med. Magnus Bell, Andernach/ M Worm, Department of Dermatology, Allergy and Venereology, Charité Berlin/ C Handrick, Practice Dr. med. Christiane Handrick, Berlin/ T Schirmer, Practice Dr. med. Thomas Schirmer, Berlin/ J Rossbacher, Practice Jens Rossbacher/ Dr. med. Klaus Spickermann, Hautzentrum, Friedrichshain/ T Bieber, Department of Dermatology and Allergology, University Hospital Bonn/ U Schwichtenberg, Practices Derma-Nord, Bremen/ K Neubert, Practice Dipl.-Med. Kathrin Neubert, Burgstaedt/ B Gerlach, Practice Dr. med. Beatrice Gerlach, Dresden/ U Boashi, Practice Dr. med. Ute Boashie, Dresden/ B Homey, Department of Dermatology and Allergology, University Hospital Duesseldorf/ M Mempel, Practice Prof. Dr. med. Martin Mempel, Elmshorn/ M Sticherling, Department of Dermatology, University, German Center for Immunotherapy, Erlangen/ SH Hong-Weldemann, Practice Dr. med. Sung-Hei Hong-Weldemann, Freiburg/ E Tchitcherina, Practice for skin and venereal diseases, Friedberg/ P Buck, Practice Dr. med. Philipp Buck, Goldbek medical, Hamburg/ M Augustin, Institute for Health Services Research in Dermatology Hamburg, University Medical Center Hamburg Eppendorf/ M Pawlak, Practice Dr. med. Anika Huenermund and Mario Pawlak, Heiligenstadt/ T Schaefer, Practice Dr. med. Thomas Schaefer/ Dr. med. Doreen Belz, Derma Koeln, Koeln/ B Schwarz, Practice Dr. med. Beate Schwarz, Langenau/ P Staubach-Renz, Clinic for Dermatology, University Hospital, Mainz/ T Biedermann, Department of Dermatology and Allergy, School of Medicine, Technical University of Munich/ F Schenck, Dermatology Center, Hannover/ M Stahl, Practice Dr. med. Maren Stahl, Osterode/ R von Kiedrowski, Focus Practice for chronic inflammatory dermatoses, skin cancer and allergology and also Study Center CMS3 (Company for Medical Study and Service), Selters/Westerwald.

**Supplementary Tables**

Table S1. Previous research on eczema and hypertensive drug intake.

| Type | Sample | Drugs | Result | Eczema Type | Reference |
| --- | --- | --- | --- | --- | --- |
| Retrospective study (exploratory)^1^ | 83 patients aged 65+ | All | Established a link between Eczematiform eruption and drug intake | Eczematiform eruption | Morin et al. 2002 Ann Derm Venerol |
| Medsafe reports^2^ | Case reports in New Zealand | CCBs (Amlodipine, Felodipine) | Two cases of adult-onset eczema linked to CCB use; insufficient data for definitive conclusions. | Eczematous dermatitis | Medsafe report 2004 |
| French Case-Control Study^3^ | 102 patients aged 60+ with 204 matched controls | CCBs | OR 2.50 (95% CI: 1.30–4.60) for eczematous dermatitis after CCB use. | Eczematous dermatitis | Joly et al. 2007 JID |
| U.S. Case-Control Study^4^ | 94 patients aged 50+ with 132 matched controls | CCBs, Thiazides | OR 4.21 (95% CI: 1.77–9.97) for CCBs; OR 2.07 (95% CI: 1.08–3.96) for thiazides. | Eczematous rashes | Summers et al. 2013 JAMA Derm |
| UK cohort study^5^ | 1.5 million older adults in the UK, aged 60+ | Diuretics, CCBs, ACE inhibitors | Diuretics (HR 1.21) and CCBs (HR 1.16) had the highest risk; ACE inhibitors (HR 1.02) and beta-blockers had the lowest risk. | Eczematous dermatitis | Ye et al. 2024 JAMA Derm |

CCBs = calcium channel blockers, CI = Confidence Interval, HR = Hazard ratio, OR = Odds ratio

Other published data includes one review ^6^ and two case reports^7,8^.

References

1. Morin C, Joly P, Courville P, et al*.* [Chronic eczematiform eruption in the elderly]. *Ann Dermatol Venereol.* 2002;129(1 Pt 1):19-22.

2. Calcium channel blockers and the possible risk of new-onset eczema. https://www.medsafe.govt.nz/safety/Alerts/Calcium_channel_blockers_and_possible_risk_of_new_onset_eczema.asp.[ Last accessed 8 Apr, 2025].

3. Joly P, Benoit-Corven C, Baricault S, et al*.* Chronic eczematous eruptions of the elderly are associated with chronic exposure to calcium channel blockers: results from a case-control study. *J Invest Dermatol.* 2007;127:2766-71.

4. Summers EM, Bingham CS, Dahle KW, et al*.* Chronic eczematous eruptions in the aging: further support for an association with exposure to calcium channel blockers. *JAMA Dermatol.* 2013;149:814-8.

5. Ye M, Chan LN, Douglas I, et al*.* Antihypertensive Medications and Eczematous Dermatitis in Older Adults. *JAMA Dermatol.* 2024;160:710.

6. Tétart F, Joly P. Eczema in elderly people. *Eur J Dermatol.* 2020;30:663-7.

7. Yoo J, Jue M-S. Intractable pruritus with chronic eczema in an elderly patient caused by long-term intake of calcium channel blocker. *Contact Dermatitis.* 2017;77:339-40.

8. Abdelwahab R, Tangalos EG, Matulis J. Differentiation of hydrochlorothiazide-induced dermatitis from stasis dermatitis. *BMJ Case Rep.* 2022;15:e249884.
